# Spermatogenesis associated serine rich 2 like plays a prognostic factor and therapeutic target in acute myeloid leukemia by regulating the JAK2/STAT3/STAT5 axis

**DOI:** 10.1186/s12967-023-03968-0

**Published:** 2023-02-11

**Authors:** Fenglin Li, Wenle Ye, Yiyi Yao, Wenwen Wei, Xiangjie Lin, Haihui Zhuang, Chenying Li, Xia Li, Qing Ling, Chao Hu, Xin Huang, Yu Qian, Shihui Mao, Jiansong Huang, Ying Lu, Jie Jin

**Affiliations:** 1 Department of Hematology, The First Affiliated Hospital, College of Medicine, Zhejiang University, Qingchun road 79#, Hangzhou, China; 2 Department of Hematology, The Affiliated People’s Hospital of Ningbo University, Baizhang road 251#, Ningbo, China; 3 Zhejiang Provincial Key Lab of Hematopoietic Malignancy, Zhejiang University, Hangzhou, China; 4 Zhejiang University Cancer Center, Hangzhou, China

**Keywords:** SPATS2L, AML, Growth, Apoptosis, JAK/STAT pathway, Prognostic, Therapeutic target

## Abstract

**Background:**

Spermatogenesis associated serine rich 2 like (SPATS2L) was highly expressed in homoharringtonine (HHT) resistant acute myeloid leukemia (AML) cell lines. However, its role is little known in AML. The present study aimed to investigate the function of SPATS2L in AML pathogenesis and elucidate the underlying molecular mechanisms.

**Methods:**

Overall survival (OS), event-free survival (EFS), relapse-free survival (RFS) were used to evaluate the prognostic impact of SPATS2L for AML from TCGA database and ourcohort. ShRNA was used to knockdown the expression of SPATS2L. Apoptosis was assessed by flow cytometry. The changes of proteins were assessed by Western blot(WB). A xenotransplantation mice model was used to evaluate in vivo growth and survival. RNA sequencing was performed to elucidate the molecular mechanisms underlying the role of SPATS2L in AML.

**Results:**

SPATS2L expression increased with increasing resistance indexes(RI) in HHT-resistant cell lines we had constructed. Higher SPATS2L expression was observed in intermediate/high-risk patients than in favorable patients. Meanwhile, decreased SPATS2L expression was observed in AML patients achieving complete remission (CR). Multivariate analysis showed high SPATS2L expression was an independent poor predictor of OS, EFS, RFS in AML. SPATS2L knock down (KD) suppressed cell growth, induced apoptosis, and suppressed key proteins of JAK/STAT pathway, such as JAK2, STAT3, STAT5 in AML cells. Inhibiting SPATS2L expression markedly enhanced the pro-apoptotic effects of traditional chemotherapeutics (Ara-c, IDA, and HHT).

**Conclusions:**

High expression of SPATS2L is a poor prognostic factor in AML, and targeting SPATS2L may be a promising therapeutic strategy for AML patients.

**Supplementary Information:**

The online version contains supplementary material available at 10.1186/s12967-023-03968-0.

## Introduction

Acute myeloid leukemia (AML) is the most common acute leukemia in adults and is characterized by the accumulation of immature myeloid blasts in the bone marrow or peripheral blood (PB) [1]. Despite progress in treatment and management strategies, AML still has a high relapse rate and a dismal long-term OS [2, 3]. Recently, an increasing number of molecular targets have been involved in leukemia development and chemotherapy resistance [4]. With the advent of novel assay technologies strategies, targeting these biomarkers has been consecutively attempted in fields including the diagnosis, prognosis, monitoring, and targeted treatment of AML [5–8]. Nevertheless, exploring and confirming new prognostic and/or therapeutic markers for AML are urgently required.

SPATS2L (synonyms: SPS2L, SGNP, and DNAPTP6) was one of the top 50 upregulated differentially expressed genes (DEGs) in HHT-resistant cell lines, which were established by our research group. Additionally, SPATS2L had prognostic significance for AML compared with other top 50 DEGs when analyzing online databases (UALCAL [9] and GEPIA). Thus, we speculated that SPATS2L played a crucial role in the pathogenesis of AML. SPATS2L was first identified by Zhu et al. in a study of oxidative stress in human myoblasts in 2008 [10]. Through genome-wide association analysis (GWAS), RNA-sequence analysis (RNA-seq), and microarray analysis, SPATS2L was identified as a location of SNPs or a DEG in asthma [11], schizophrenia [12], hypertension [13], acute respiratory distress syndrome [14], skeletal muscle cell differentiation [15], psoriasis [16], diabetes [17], and chronic abdominal pain [18]. However, a few of these results were confirmed by further experiments. SPATS2L remains poorly investigated in its location, function, and mode of action in hematologic neoplasms.

In the present study, we aimed to identify the location and expression characteristics of SPATS2L, investigate its prognostic significance, and evaluate its functions and molecular mechanisms in AML. We also explored whether suppressingSPATS2L expression could enhance the anti-leukemic effect of chemotherapy drugs, Cytarabine (Ara-c), Idarubicin (IDA), and HHT in AML cells. We hypothesized that SPATS2L was an independent prognostic factor in AML and might represent a novel therapeutic target for AML patients.

## Materials and methods

### Patients and cell culture

AML cell lines THP-1, HL-60, OCI-AML3, NB4, Kasumi-1, KG-1, U937, THP-1-luc, were cultured in RPMI medium (Corning Cellgro, USA), MV4-11(MV4-11S), HHT resistance MV4-11 cell lines (MV4-11R10, R30, R50), MOLM13(MOLM13 S), HHT resistance MOLM13 cell lines (MOLM13 R10, R30, R50) were cultured in IMDM medium (Corning Cellgro, USA). The medium was supplemented with 10% fetal calf serum (FCS), penicillin G (100 unit/mL), and streptomycin (100 μg/mL) at 37 °C in a 5% CO2 atmosphere. HEK-293 T cells were cultured in DMEM medium supplemented with 10% fetal bovine serum (Thermo Fisher Scientific, Gibco, USA) at 37 °C with 5% CO2.

A total of 228 adult de novo AML patients with detailed diagnosis and treatment information were included in this study from March 2010 to June 2016 according to standard French–American–British and World Health Organization criteria between 2003 and 2011. Patients with acute promyelocytic leukemia (APL) and those undergoing bone marrow transplantation were excluded. WHO classification, conventional cytogenetic banding assays, and molecular analyses were performed centrally at the Zhejiang Provincial Key Lab of Hematopoietic Malignancy, China, as previously described [19]. We analyzed the chromosomal abnormalities and gene mutations of NPM1, FLT3-ITD, CEBPA, DNMT3A, IDH1and IDH2 using previously described methods [20]. Before performing the above cytogenetics analyses, the SPATS2L expression profile and clinical outcome were unknown. This study was approved by the Research Ethics Committee of the First Affiliated Hospital, College of Medicine, Zhejiang University. All the patients provided written informed consent to participate in the study.

### Construction of HHT resistant cell lines with increasing resistance index (RI)

MV4-11 and MOLM13 cells were treated with gradually increasing HHT concentrations (1 nmol/L–50 nmol/L). Cells that grew normally in an IMDM medium with 10% fetal bovine containing 10 nmol/L, 30nml/L, and 50 nmol/L HHT were preserved. These cells were named MV4-11 R10, MV4-11 R30, MV4-11 R50(MV4-11 R), MOLM13 R10, MOLM13 R30, and MOLM13 R50. Then, the IC50 value of those cells was calculated. The RI of those cells was calculated with the formula IC50 (Resistance)/IC50 (Sensitive).

### Drugs

HHT (#61847) was purchased from MCE (MedChemExpress, USA). Ara-c (# S1648), and IDA HCl (# S1228) were purchased from Selleck (Shanghai, China).

### Quantitative real-time PCR (qRT-PCR)

Total RNA was extracted using TRIzol reagent (Takara, Japan). First-strand complementary DNA synthesis was performed using the PrimeScript^™^ IV 1st strand cDNA Synthesis Mix (Takara, Japan). Quantitative PCR was performed in triplicate using a SYBR Green PCR Master Mix kit (Takara, Japan) on an IQ5 real-time PCR instrument (Bio-Rad, USA). The mRNA levels were normalized to GAPDH. The primers used were as follows.

SPATS2L forward:5’-AAGCAGCATCAAGGCAACAAA-3′,

reverse: 5′-TTCTCGCAGCCATTCATGGG-3′,

GAPDH forward: 5′-GGAGCGAGATCCCTCCAAAAT-3′;

reverse 5′-GGCTGTTGTCATACTTCTCATGG-3′.

### Confocal fluorescence

To explore the localization of SPATS2L in AML cells, 1 × 10^6^ cells were harvested and fixed in 4% paraformaldehyde for 15 min. Cells were then incubated with 0.3% Triton X-100 for 15 min and incubated with SPATS2L antibody at 4 ℃ overnight. Then cells were washed twice and incubated with a fluorescent secondary antibody for 1 h. After incubation with DAPI for 15 min, cells were transferred to a confocal 96-well plate and visualized using Nikon A1 Ti.

### Cell viability assay

Cells were seeded in 96-well plates at 0.5 × 10^5^ for growth or 1 × 10^5^ for drug sensitivity in each well. 20 μl MTS solutions (Promega, Madison, WI) were added to each well, and the cells were incubated for 4 h at 37 °C. The plates were read at a wavelength of 490 nm using Varioskan Flash (Thermofisher, USA).

### Flow cytometry analysis and sorting by Flow cytometry

Cells transfected with control or SPATS2L KD lentiviral fluid were collected after transfection 96 h. For apoptosis analysis, cells were stained with PI and Annexin-V-APC for 15 min in a binding buffer (MULTI SCIENCES, China). For the differentiation test, CD11b FITC (BD, USA) and CD14 FITC (BD, USA) were used. Cells were stained with CD11b or CD14 antibody for 30 min in 1xPBS. All the processed cells were analyzed using a NovoCyte D2060R flow cytometer (ACEA, China). GFP-positive cells were sorted by Beckman moflo Astrios EQ.

### Western blot analysis

Cells were harvested, and 1 × RIPA buffer containing a protease inhibitor and phosphatase inhibitor cocktail (Thermo Fisher Scientific, USA) was added on ice for 30 min and centrifuged at 12,000 g for 15 min at 4 ℃ to pellet cell debris. Protein concentration was determined using a BCA reagent (Thermo Fisher Scientific, USA). After heating to 100 ℃ for 10 min, the protein samples were separated by SDS-PAGE and transferred to PVDF membranes (Millipore, Burlington, MA, USA). The membrane was blocked with 5% powder milk and probed with an antibody at 1:1000. After washing with 1 × TBST buffer, the membrane was incubated with HRP-conjugated secondary antibodies at 1:5000. The target proteins were visualized using an ECL kit (Thermo Fisher Scientific, USA) and imaged using a Bio-Rad imaging system. Antibodies against GAPDH #8884, STAT3 #4904, STAT5 #94205S, Phospho-STAT5 #9359, Phospho-BCL2 #2827S, Caspase3 #9662S, PARP #9532S were purchased from CST (Danvers, MA). Antibodies against SPATS2L 16938-1-AP, JAK2 17670-1AP, BCL2 26593-1-AP were purchased from ProteinTech (Rosemont, USA). The antibody against CD45 (PE conjugated) was purchased from MULTI SCIENCES (Hangzhou, China).

### RNA-sequence and RNA-seq analysis

RNA-sequencing expression (level 3) profiles and corresponding clinical information for AML were downloaded from the TCGA dataset(https://portal.gdc.com). R packages were implemented by R (foundation for statistical computing 2020) version 4.0.3. The mRNA expression profiles of HHT paternal strains (MV4-11 S) and HHT-resistant strains with different resistance indexes (RIs), MV4-11 R10, MV4-11R30, MV4-11R50, were obtained using high throughput sequencing (RNA-Seq). Sequence data were obtained from Illumina HiSeq X Ten platforms and uploaded to NCBI's SRA data (PRJNA664675). For RNA-Seq data, mRNA expression levels were calculated as RPKM (Reads Per Kilo-base per Million reads). The DE Seq (1.18.0) R package analyzed differentially expressed mRNAs. For SPATS2L KD RNA-seq, RNA was isolated from THP-1 control and THP-1 SPATS2L KD cells. The sequence was obtained from the Illumina HiSeq X Ten platform and uploaded to NCBI's SRA data (PRJNA707119). In the present study, the screening criteria for differential expression genes (DEGs) are fold-change  > 2 and P value  < 0.05.

### Gene set enrichment analysis (GSEA) and kyoto encyclopedia of genes and genomes (KEGG) analysis

GSEA version 3.0 was used to analyze and interpret RAN-sequence data according to biological knowledge [21]. SPATS2L control and KD RNA-sequence data and TCGA AML patients grouped by low and high SPATS2L expression RNA-sequence data were analyzed by GSEA using h.all.v7.2.symbols.gmt (hallmarks). Gene sets with normalized FDR  < 0.05 were selected in this study. KEGG analysis were made by oebiotech company (Shanghai,China).

### SPATS2L KD by shRNA

To knock down the expression of SPATS2L in AML cells, shRNA was performed using available sequences targeting SPATS2L and non-targeting control. The sequences of shRNA were as follows.

control 5′-ACAGAAGCGATTGTTGATC-3′,

SPATS2L sh1 5′- GGATGGCAGTGCAATTCAAGT-3′,

SPATS2L sh2 5′ GGAGAAATTACACATCCAAAG-3′.

Briefly, shRNA targeting or control plasmid with a green fluorescent protein (GFP) containing and package plasmids (psPAX2 and pMG2.G) were co-transfected into HEK-293 T cells in a 10 cm cell culture dish using Calcium Phosphate Cell Transfection Kit (Beyotime, China). Lentivirus particles were harvested at 48 h and directly added to 1 × 10^6^ cells. Infection efficiency was determined by detecting GFP expression by flow cytometry. The decrease in SPATS2L expression was confirmed by qRT-PCR and Western blot.

### CDX mice model

6–8 weeks old immunodeficient NCG(NOD/ShiLtJGptPrkdcem26Cd52Il2rgem26Cd22/Gpt) mice were raised in the Experimental Animal Center of Zhejiang Chinese Medicine University Laboratory Animal Research Center. All animal experiments were approved by the Ethics Committee of the Experimental Animal Center of Zhejiang Chinese Medicine University Laboratory Animal Research Center (Hangzhou, China). THP-1 cells were transfected with the SPATS2L control or KD lentiviral solution. After 72 h, 1 × 10^6^ GFP positive cells were injected into mice through the tail vein. Each group had six mice. Bodyweight and CD45 expression of PB were measured every week. THP-1 luc cells transfected with SPATS2L control or KD lentiviral solution were injected into mice through the tail vein to evaluate the tumor burden more intuitively. The number of the two groups was five and six, respectively. Cell engraftment was assessed by intraperitoneal luciferin injection (100 mg/kg) and then by imaging using an IVIS Lumina LT system (PerkinElmer, CA, USA). The leukemic burden of mice was assessed by bioluminescence imaging every seven days after injection and analyzed using GraphPad Prism 5 [two-tailed Student's t-tests]. Mice were euthanized when they developed a bowed back, or their lower limbs were paralyzed. The survival of each group of mice was analyzed using Kaplan–Meier curve analysis by IBM SPSS Statistics 20 software.

### Statistical analysis

The primary endpoints of this study were OS, EFS, and RFS. OS was defined as the time from diagnosis until death due to any cause or last follow-up. EFS was defined as the time to diagnosis until its removal from the study due to incomplete remission, relapse, or death. RFS was defined as the time from CR to relapse or death as previously described[19]. The survival of OS, EFS, and RES was analyzed using Kaplan–Meier curve analysis, and multivariate analysis was used cox regression method by IBM SPSS Statistics 20 software.

For experimental analysis, unpaired two-tailed Student's t-test or ANOVA and Chi-square test were used by IBM SPSS Statistics 20 software or GraphPad Prism 5. P < 0.05 was considered statistically significant. P < 0.05 *, P < 0.01 **, P < 0.001 ***.

## Results

### SPATS2L was upregulated in HHT-resistant cells

**Fig. 1:**
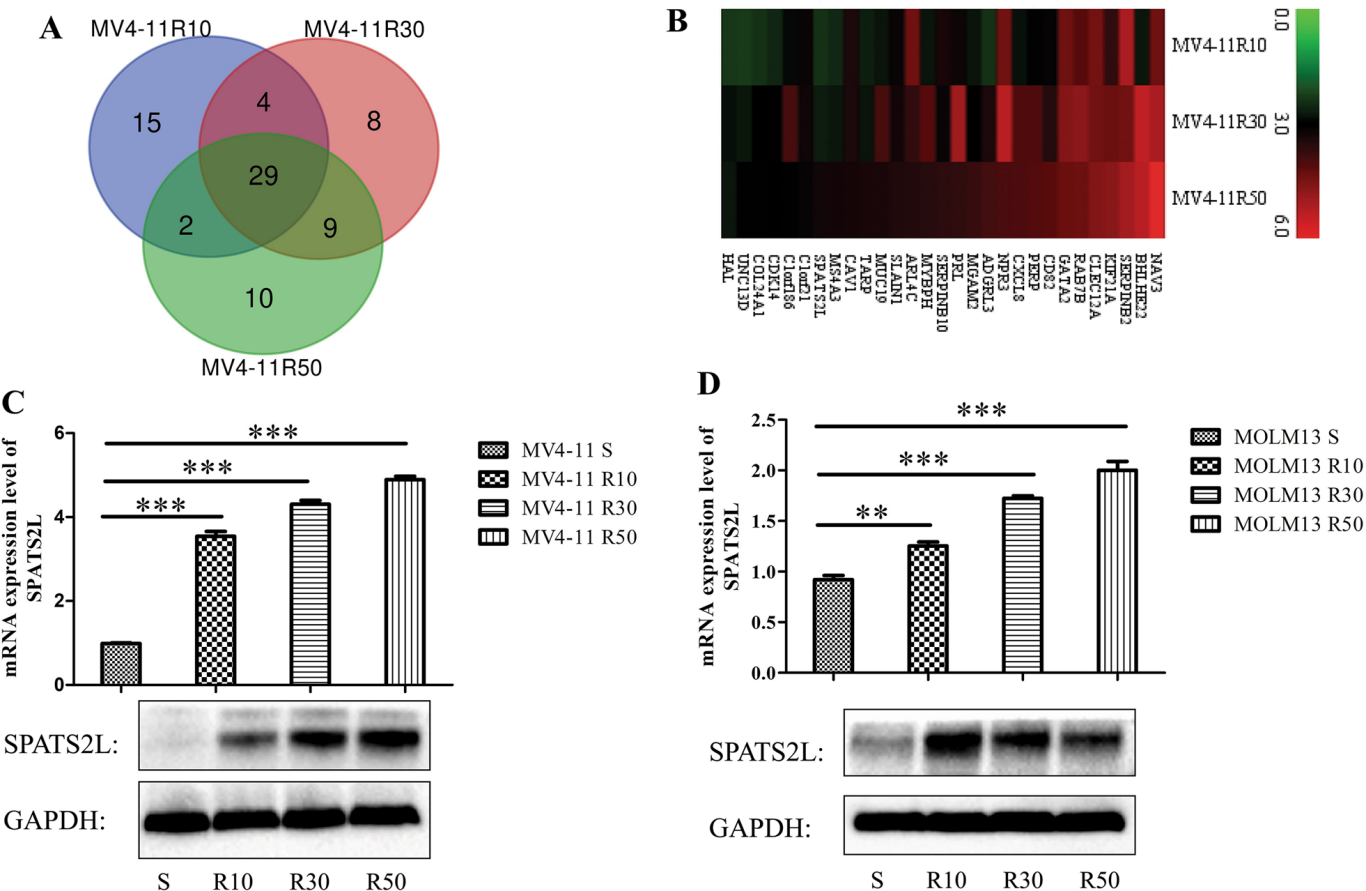
SPATS2L is upregulated in HHT-resistant cell lines. **A** Integrative analysis of top 50 up-regulated DEGs of our RNA-seq data from HHT-resistant cell lines to identify DEGs commonly shared among different HHT resistance indexes by the venn method. **B** The Log_2_ fold change of the 29 commonly upregulated DEGs. **C** The mRNA and protein expression levels of SPATS2L between HHT-sensitive and resistant cells from MV4-11 by qRT-PCR and western blot, respectively. **D** The mRNA and protein expression levels of SPATS2L between HHT-sensitive and resistant cells from MOLM13 by qRT-PCR and western blot, respectively. NS stands for not significant, P < 0.05 *, P < 0.01 **, P < 0.001 ***

To assess whether resistance occurs in AML therapeutically treated with HHT, we established 6 HHT-resistant cell lines (MV4-11 R10, MV4-11 R30, MV4-11 R50, MOLM13 R10, MOLM13 R30, and MOLM13 R50) with increasing RI. Then, to investigate the mRNA expression profile changes associated with HHT resistance, we performed RNA sequencing between MV4-11 resistant and MV4-11 (MV4-11S) cells. A Venn diagram was generated, and 29 DEGs (belonging to the top 50 DEGs) were upregulated in the three MV4-11 resistant cell lines (Fig. 1A, B and Additional file 1: Table S1).

As drug resistance was one of the main reasons for poor prognosis, these 29 DEGs were used to explore their prognostic significance for AML using the online database UALCAN (http://ualcan.path.uab.edu/) and GEPIA (http://gepia.cancer-pku.cn/). Of them, SPATS2L had the strongest prognostic significance for OS (p < 0.0001) (Table S1). Interestingly, with the increase of RI, the mRNA and protein levels of SPATS2L were also increased in MV4-11 and MOLM13 resistance cells (Fig. 1C, D). These results indicated SPATS2L expression might be related to HHT resistance and play a critical role in AML.

### High expression of SPATS2L is an independent risk factor of AML

Given that SPATS2L is little studied in cancer, including AML, we sought to identify its protein localization and expression characteristics in AML cells and patients. Confocal fluorescence results illustrated that SPATS2L was located in the nucleus of AML cells (Additional file 1: Fig. S1A). Then we analyzed the SPATS2L mRNA of 228 newly diagnosed and untreated AML patients from our cohort, and the clinical characteristics were summarized in Table 1. Higher SPATS2L expression was observed in AML patients with the M5 type (acute monocytic leukemia) than those with the other types in TCGA and our cohorts (Additional file 1: Fig. S1B). Notably, higher SPATS2L expression was found in intermediate/high-risk AML patients than in favorable-risk patients (Additional file 1: Fig. S1C). These observations corroborated the notion that SPATS2L might have prognostic significance in AML.

**Table 1:** The SPATS2L expression characteristics of AM patients between low and high group

Variables	SPATS2L expression		*P* value
	Low group	High group	
Number, n (%)	111 (50%)	111 (50%)	
Age, median(range)	46 (15,80)	52 (14,82)	0.012
Female, n (%)	45 (40.5)	56 (50.5)	0.178
WBC^1^, median(range)	21.70 (0.2,453.2)	20.8 (0.7,229.90)	0.284
HB^2^, median(range)	85.0 (33.5,141.0)	80.0 (37.4,135)	0.090
PLT^3^, median(range)	33 (2,556)	62 (4,778)	0.001
BM^4^ blast, median(range)%	70 (20,98)	69 (17.5,96.65)	0.810
FAB^5^ type			0.037
M0	8 (7.2)	13 (11.7)	
M1	17 (15.3)	8 (7.2)	
M2	53 (47.7)	40 (36.0)	
M4	6 (5.4)	5 (4.5)	
M5	23 (20.7)	40 (36)	
M6	2 (1.8)	4 (3.6)	
Karyotype risk^6^, n (%)			0.154
Favorable	3 (2.7)	2 (1.8)	
Intermediate	96 (80.5)	104 (93.7)	
Unfavorable	7 (16.3)	2 (1.8)	
Gene mutation			
FLT3-ITD	18 (16.2)	26 (23.4)	0.239
NPM1	24 (21.6)	38 (34.2)	0.052
CEBPA^DM7^	19 (17.1)	13 (11.7)	0.122
IDH1	5 (4.5)	14 (12.6)	0.053
IDH2	9 (8.1)	12 (10.8)	0.647
DNMT3A	7 (6.3)	16 (14.4)	0.076
Treatment protocols^8^			0.352
DA or IA	82 (76.6)	73 (70.2)	
HAA	25 (23.4)	30 (28.8)	
Clinical event			
Complete remission rate (%)	85 (81.7)	74 (73.3)	0.181
Relapse rate (%)	21 (25)	39 (52.7)	0.001
Death rate (%)	41 (36.9)	57 (52.3)	0.03

^1^WBC, white blood cell; ^2^HB, hemoglobin; ^3^PLT, platelet counts; ^4^BM, bone marrow; ^5^FAB, French American British classification systems. ^6^Karyotype Risk, Favorable subgroup comprised t(8;21)/AML1-ETO, inv16 or t(16;16)(p13.1;q22)/CBFb-MYH11,CEPRA^DM^, NPM1 without FLT3-ITD or FLT3-ITD^low^. Adverse group consisted of t (9;22), inv(3)/t(3;3), -5, -7, del (5q), del(7p), 11q23 and complex translocations. Intermediate subtype contained cytogenetically normal and AML with other cytogenetic abnormalities. ^7^DM: Double-allele. ^6^The protocols used for induction therapy in different groups including donorubicin/Ara-C (DA)-based treatment group, idarubicin/Ara-C (IA)-based, and homoharringtonine/Ara-C/aclarubicin (HAA)-based treatment group

Additionally, SPATS2L expression was significantly decreased in primary AML patients when achieving CR (Additional file 1: Fig. S1D), and higher SPATS2L protein levels were frequently observed in AML cells than in normal cells (Additional file 1: Fig. S1E). These observations revealed that SPATS2L might have a treatment response in AML and could be a therapeutic target.

**Fig. 2:**
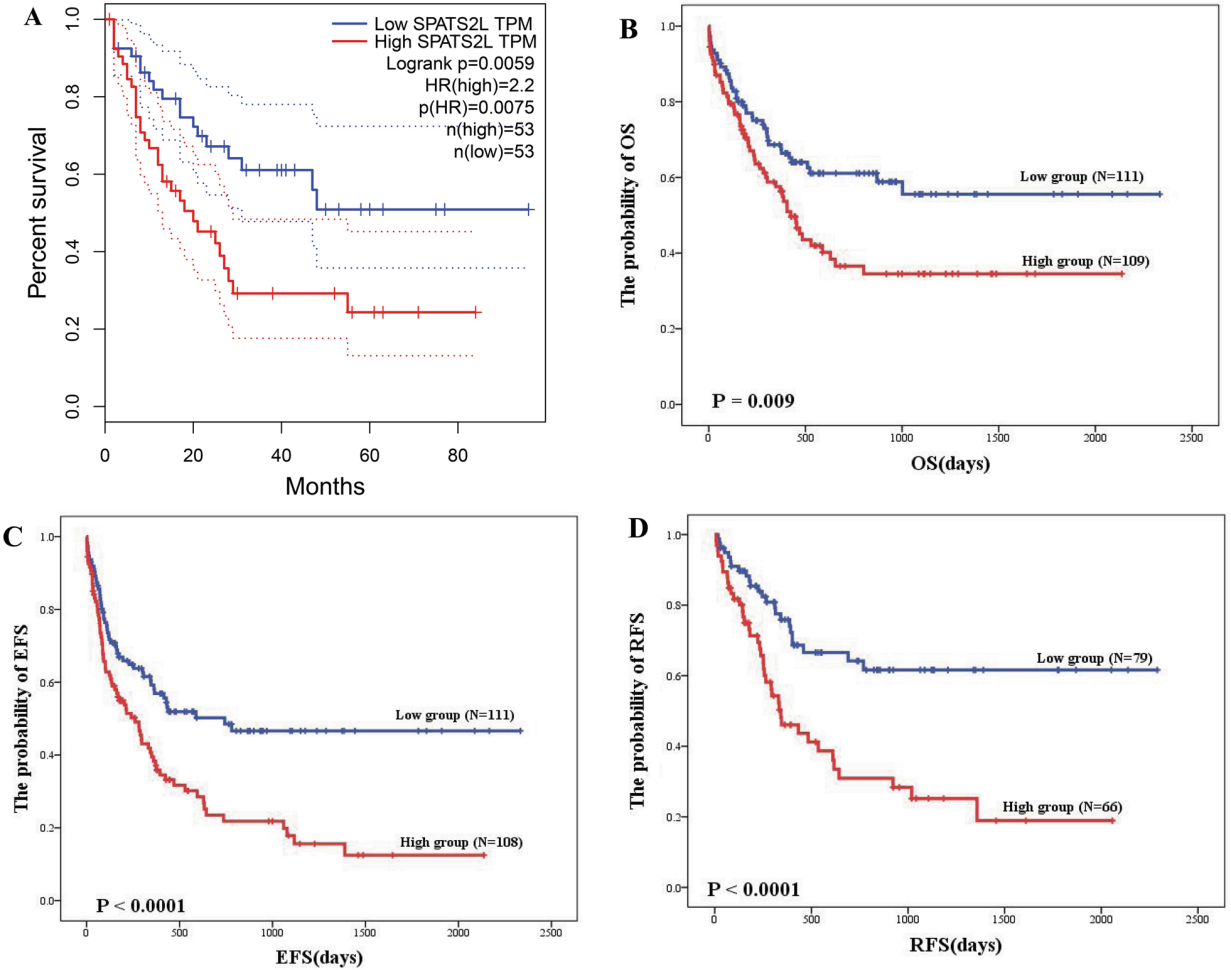
The prognostic significance of SPATS2L in AML. **A** OS of SPATS2L low (blue) group and the high (red) group from GEPIA database (log-rank tests; P-value = 0.0059)(http://gepia.cancer-pku.cn/). **B** OS of SPATS2L low (blue) group and the high (red) group from our cohort (log-rank tests; P-value = 0.009). **C** EFS of SPATS2L low (blue) group and the high (red) group from our cohort (log-rank tests; P-value < 0.0001). **D** RFS of SPATS2L low (blue) group and the high (red) group from our cohort (log-rank tests; P-value < 0.0001)

Two independent cohorts were utilized for survival analysis to further confirm whether SPATS2L could be a prognostic marker for AML. The public database GEPIA showed AML patients with high SPATS2L expression had a shorter OS (Fig. 2A). Subsequently, data from our center were used to validate the prognostic significance with the different clinical endpoints. AML patients were divided into high and low groups based on the median value of SPATS2L expression according to quartile survival analysis (Additional file 1: Fig. S2A–C). Survival analysis showed that patients with high SPATS2L expression had a shorter OS (P = 0.009) (Fig. 2B), which was consistent with the public database results. In addition, higher SPATS2L expression resulted in worse EFS (P < 0.0001) (Fig. 2C) and RFS (P < 0.0001) (Fig. 2D).

Further multivariate analysis demonstrated high SPATS2L expression in AML was an independent poor prognostic marker for OS (P = 0.025, HR = 1.631), EFS (P = 0.0001, HR = 1.894), and especially for RFS (P < 0.0001, HR = 3.345) (Table 2). These results demonstrated that high SPATS2L expression was an independent marker of poor prognosis for AML and had a stronger prognostic significance for RFS than for OS and EFS.

**Table 2:** Multivariate analysis of the prognostic significance of SPATS2L on AML from our cohort

Clinical endpoint	Risk factor*	P value	HR(95%CI)
Overall survival(OS)	WBC	0.000	1.007 (1.004,1.010)
	HB	0.039	.0990 (0.981,0.999)
	Blast	0.053	1.011 (1.000,1.022)
	CEBPa	0.006	0.571 (0.381,0.851)
	Age	0.001	1.022 (1.008,1.036)
	SPATS2L	0.025	1.631 (1.064,2.500)
Event free survival(EFS)	WBC	0.000	1.007 (1.005,1.010)
	HB	0.041	0.991 (0.983,1.000)
	CEBPa	0.001	0.592 (0.432,0.812)
	DNMT3A	0.053	1.644 (0.994,2.721)
	Sex	0.031	1.513 (1.039,2.202)
	SPATS2L	0.001	1.894 (1.288,2.786)
Relapse free survival(RFS)	WBC	0.001	1.007 (1.003,1.011)
	CEBPa	0.105	0.720 (0.485,1.071)
	Flt3-ITD	0.064	1.928 (0.963,3.861)
	IDH2	0.020	0.085 (0.011,0.676)
	Age	0.067	1.011 (0.999,1.034)
	SPATS2L	0.000	3.345 (1.883.5.943)

Multivariate analysis were performed by Cox proportional hazards models with the backward likehood stepwise procedures, risk factors that are not in the cox regression analysis equation are not shown in the Table2

*HR* hazard ratio, *CI* confidence interval

^*^: As for multivariate analysis, the risk factor included age,sex,WBC,HB,PLT,BM blast,FAB type, Karyotype Risk, gene mutations (FLT3-ITD,NPM1,CEBPA,IDH1,IDH2,DNMT3A), treatment protocols

### SPATS2L KD suppressed AML cell growth in vitro and prolonged survival of AML NCG mice in vivo

**Fig. 3:**
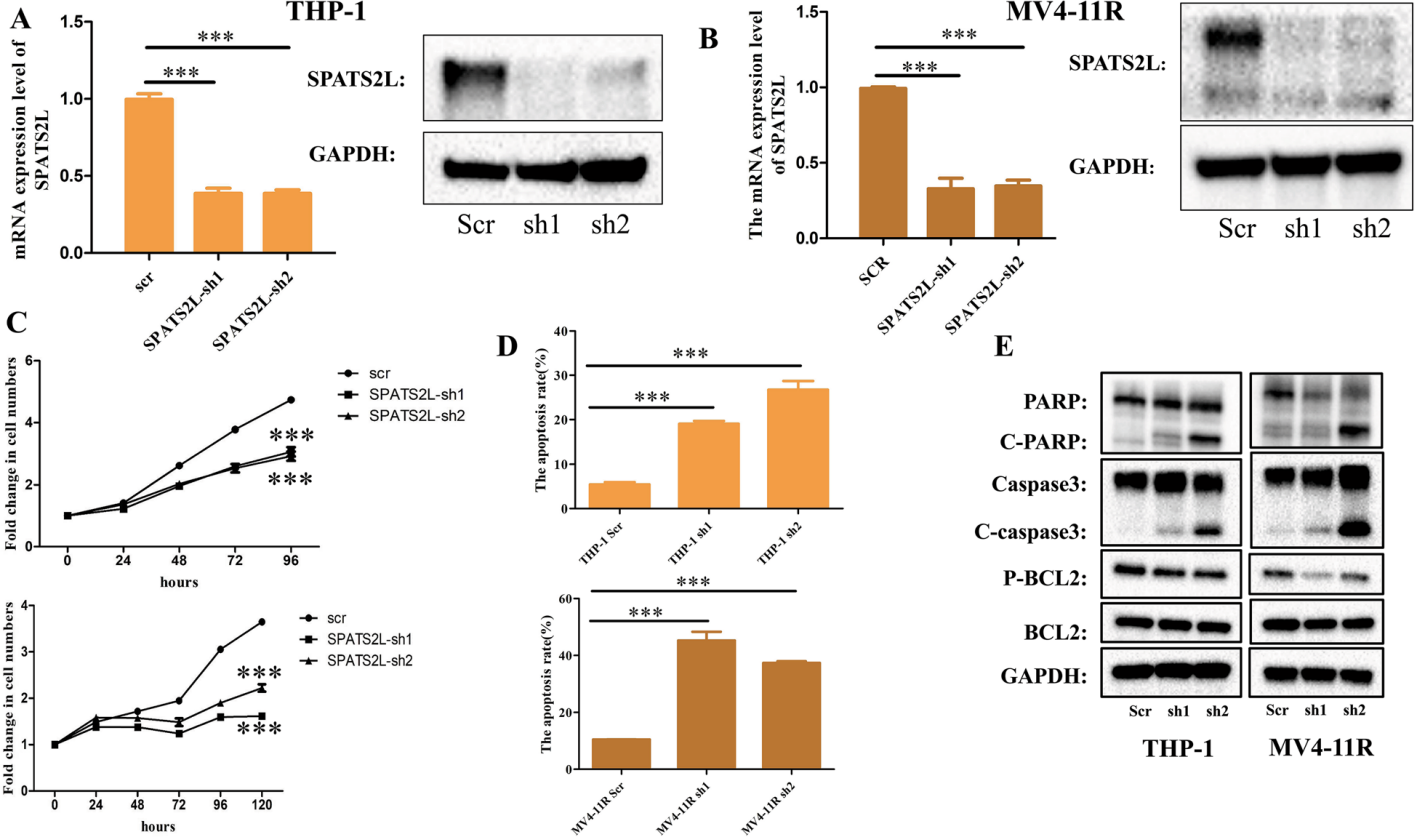
The regulating function of SPATS2L in AML. **A**–**B**. qRT-PCR and western-blot confirmed the decreased level of SPATS2L in AML cells by shRNA targeting KD. **C** The growth curve of AML cells with or without SPATS2L KD was measured by MTS. **D**. Flow-cytometry measured the apoptosis of AML cells with or without SPATS2L KD. **E**. Apoptosis-related proteins were measured by western-blot. NS stands for not significant,, P < 0.05 *, P < 0.01 **, P < 0.001 ***

Next, we sought to investigate the function of SPATS2L in AML by analyzing the effect of SPATS2L KD on the growth and viability of AML cells in vitro. The expression rate of GFP fluorescence and the mRNA and protein levels of SPATS2L confirmed the inhibitory effect of SPATS2L KD (Additional file 1: Fig. S3A–B, Fig. 3A–B). After SPATS2L KD, cell growth was suppressed (Fig. 3C), and cell apoptosis was induced in THP-1 and MV4-11R cells (Fig. 3D). However, SPATS2L KD had no impact on the cell cycle (Additional file 1: Fig. S3C–D). Only SPATS2L sh2 could increase the expression of differentiation antigens CD11b and CD14. (Additional file 1: Fig. S3E). In addition, SPATS2L KD promoted the cleavage of two apoptosis regulatory proteins PARP and caspase3, and inhibited the expression of phosphor-BCL2 without affecting total BCL2 expression (Fig. 3E). These findings indicated that SPATS2L might be a therapeutic target in AML.

**Fig. 4:**
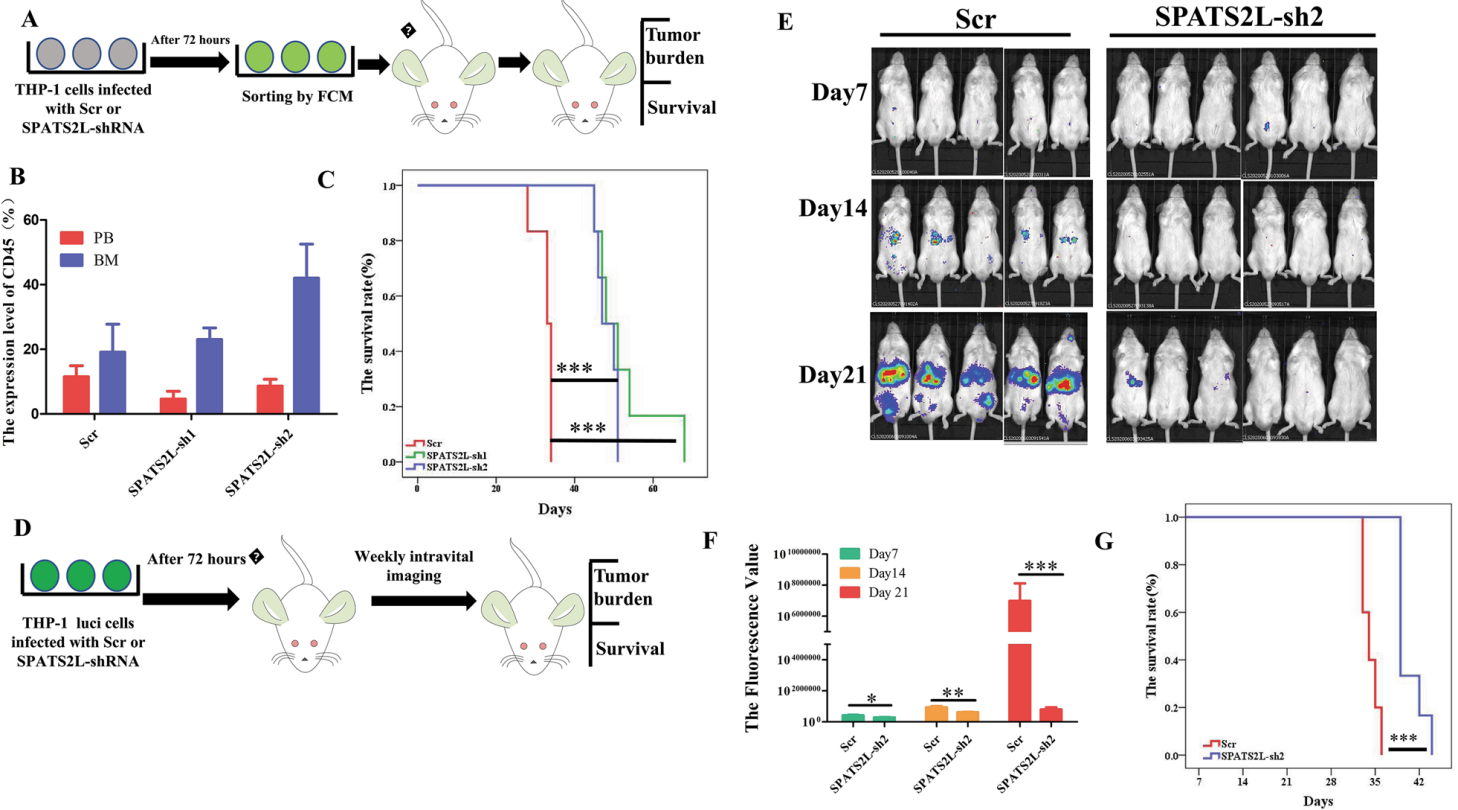
The inhibition of SPATS2L in AML can prolong the survival of AML mice. **A** Schematic illustration of the THP-1 AML xenograft NCG model coupled with control and shRNA targeting SPATS2L KD. **B** The human CD45 expression of PB and BM cells for the last time in mice was measured by flow cytometry. **C** The survival curves of the three groups. **D** Schematic illustration of the THP-1luc AML xenograft NCG model coupled with a control or shRNA targeting SPATS2L KD. **E** In vivo imaging results of the abdomen of mice on the 7th, 14th, and 21st day after cell injection. **E** Quantitative analysis results of in vivo imaging results of the abdomen of mice. F Kaplan–Meier curves of the three groups xenotransplanted with human THP-1 luc AML cells. NS not significance, P < 0.05 *, P < 0.01 **, P < 0.001 ***

To further confirm whether SPATS2L plays an essential regulatory role in AML, in vivo experiments were performed. The experimental design is shown in Fig. 4A. THP-1 cells were successfully transfected with SPAST2L control, SPATS2L-sh1, or SPATS2L-sh2 lentivirus. The level of humanized CD45 in the peripheral blood and bone marrow of mice verified the successful establishment of the AML CDX mice model (Fig. 4B). The SPATS2L sh1 and SPATS2L sh2 groups had longer survival times than the control group (Fig. 4C). To observe the tumor burden more intuitively, THP-1 with luciferase was used to determine whether SPATS2L expression could affect the tumor burden in mice (Fig. 4D). The results showed SPAST2L control group had a higher tumor burden and shorter survival than the SPATS2L KD groups (Fig. 4E–G). Altogether, these animal experiments suggest that SPATS2L inhibition could suppress tumor growth and prolong the survival of AML mice.

### SPATS2L KD suppressed JAK2/STAT3/STAT5 pathway

**Fig. 5:**
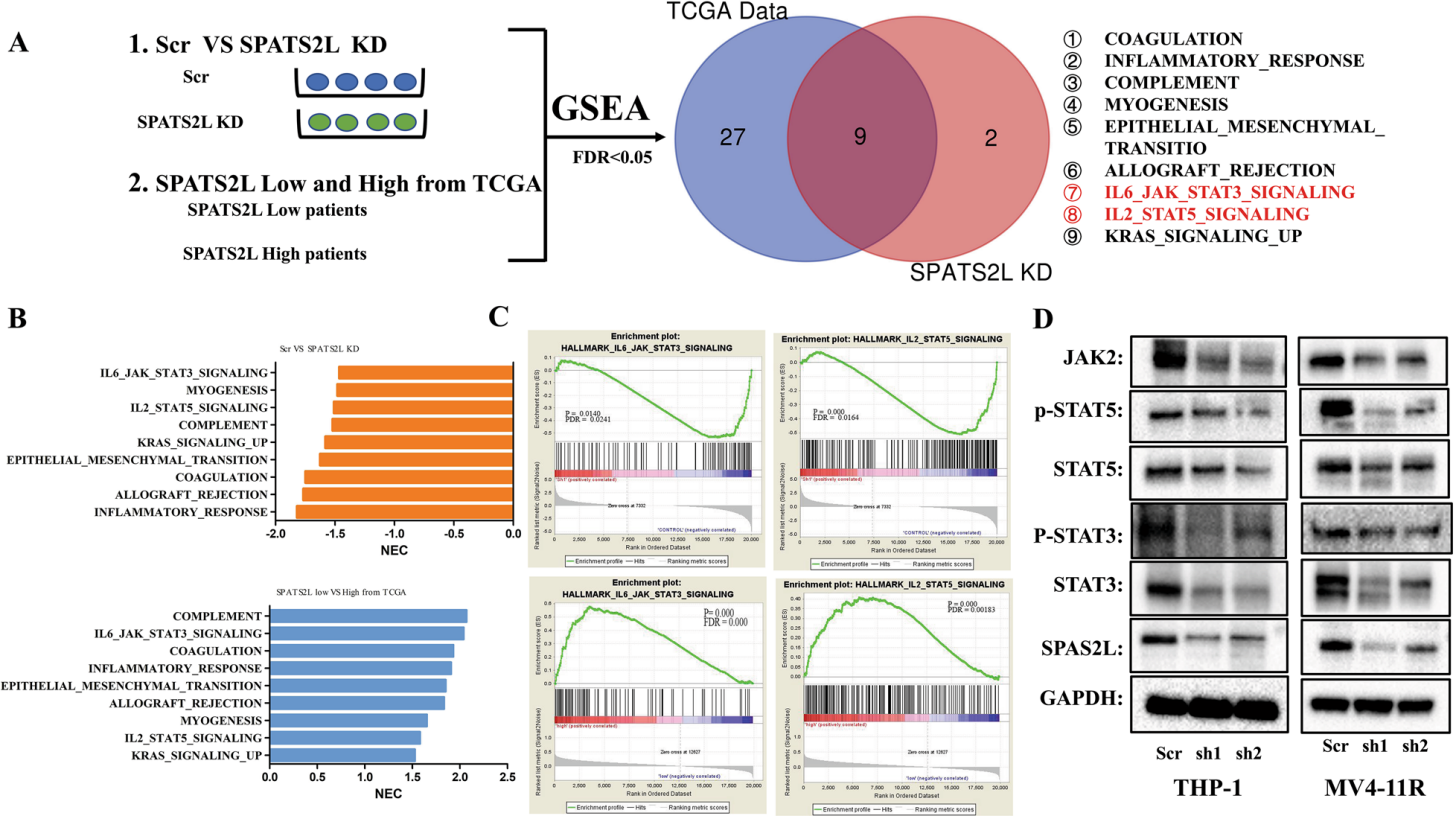
The regulating mechanism of SPATS2L in AML. **A** Integrative analysis of our SPTS2L KD RNA-seq data and TCGA AML RNA-seq data was performed to identify pathways or gene sets commonly enriched by GSEA. Nine gene sets were identified to be affected by both our and TCGA data. **B** Normalized enrichment scores (NES) of the six gene sets. **C** Among the nine signaling pathways, IL6-JAK-STAT3-SIGNALING and IL2-STAT5-SIGNALING were significantly suppressed, and the two pathways were most correlated with AML. **D** The changes of proteins JAK2/STAT3/STAT5 were measured by western-blot

To elucidate the molecular mechanisms of SPATS2L in AML, we analyzed global changes in gene expression in THP-1 cells transfected with sh-SPATS2L or control and AML patients from TCGA datasets. The RNA sequencing results showed 110 up-regulated DEGs and 355 down-regulated DEGs after SPATS2L KD in THP-1 cells (Additional file 1: Fig. S4A, B). AML patients were divided into high and low SPATS2L expression groups according to the median counts (the value of the median counts was 186) of SPATS2L. The expression profiles between the high and low patients were considerably divergent (Additional file 1: Fig. S4C). GSEA revealed that 11 gene sets from the cell line data and 36 gene sets from TCGA data were enriched with normalized FDR < 0.05, and 9 gene sets were both enriched in two cohorts (Fig. 5A). Two of the 9 gene sets belonged to JAK/STAT pathway, which involved in IL6-JAK-STAT3 signaling and IL2-STAT5 signaling, respectively. The results from GSEA indicated that these two signaling pathways were activated in SPATS2L control cells and in SPATS2L high expression patients (Fig. 5B, C). The JAK/STAT pathway is activated in AML and is considered an essential regulatory pathway in AML [22]. Therefore, we speculated that SPATS2L might function as a critical JAK/STAT signaling regulator in AML. After validation by western blot, we confirmed that SPATS2L inhibition in AML cells decreased the expression of JAK2, STAT3, and STAT5 in AML cells (Fig. 5D). To explore how SPATS2L influenced the JAK/STAT pathway, we analyzed the 355 down-regulated DEGs of THP-1 cells after SPATS2L KD by KEGG. The top 20 down-regulated gene sets were summed in Table S2 and shown in Additional file 1: Fig. S5A. Through the KEGG map, chemokine signaling pathways were suppressed after SPATS2L KD. Inhibition of cytokine-cytokine receptor interaction further inhibited the JAK/STAT signaling. Then JAK/STAT signaling influenced cell survival, growth, apoptosis, migration, differentiation, and cytokine production (Additional file 1: Fig. S5B).

### SPATS2L KD enhanced the apoptosis-promoting effect of traditional chemotherapeutics

**Fig. 6:**
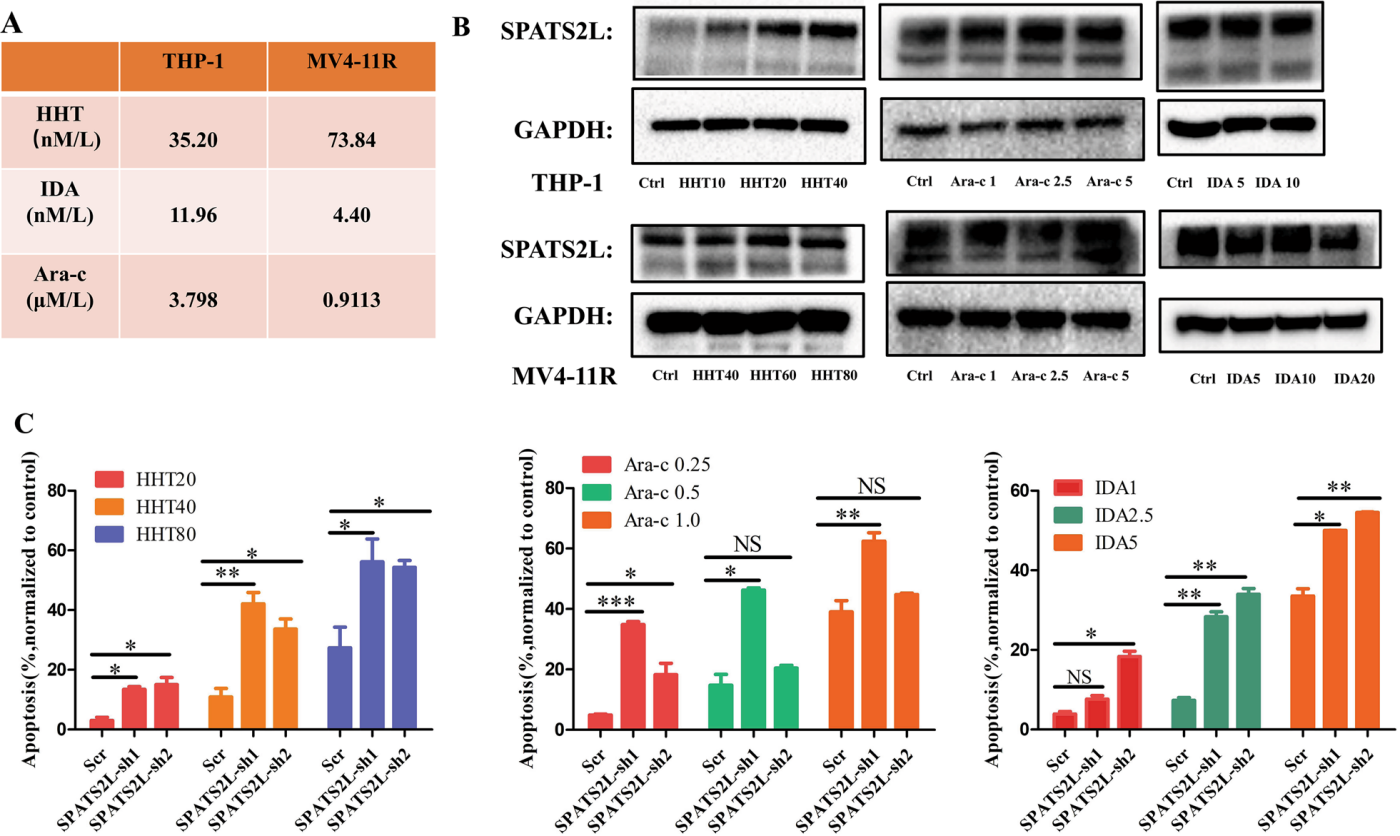
SPATS2L knockdown enhanced the apoptosis-promoting effect of traditional chemotherapeutics. **A** IC50 of AML cell lines, THP-1, MV4-11R treated by Ara-c(μM/L), IDA(nM/L), HHT(nM/L). **B** Protein level of SPATS2L in AML cells treated by Ara-c(μM/L), IDA(nM/L), HHT(nM/L). **C** Apoptosis of AML cells from THP-cell line among THP-1 scr and THP-1 SPATS2L-sh1, THP-1 SPTS2L-sh2 treated by Ara-c(μM/L), IDA(nM/L), HHT(nM/L), respectively. NS stands for not significant, P < 0.05 *, P < 0.01 **, P < 0.001 ***

As SPATS2L was an up regulated DEG in HHT-resistant cell lines, we then evaluated the function of SPATS2L in traditional AML chemotherapeutics. Disappointingly, three chemotherapy drugs, Ara-c, IDA, and HHT, did not affect the expression of SPATS2L (Fig. 6A, B). But inhibiting SPATS2L expression enhanced the apoptosis-promoting effect of these three traditional chemotherapeutics on THP-1 cells (Fig. 6C). These findings suggested that targeting SPATS2L could be a promising therapeutic option for AML patients who are resistant to chemotherapy.

## Discussion

In the present study, we first confirmed high SPATS2L was an independent marker of poor prognosis for OS, EFS, and RES in AML. Inhibition of SPATS2L in AML cells could suppress cell growth, induce cell apoptosis, suppress JAK2, STAT3, and STAT5 expression, and enhance the pro-apoptotic effects of AML chemotherapy drugs. These results indicated that SPATS2L could be a promising therapeutic target for AML treatment in the future.

The HHT-based “HAA” program (HHT plus aclarubicin and cytarabine) for AML treatment was first proposed by our group and is the first-line therapy for non-elder AML patients in China [23, 24]. With the widespread use of HHT, drug resistance has become a new challenge. As one of the top 50 upregulated genes among all resistant cell lines, the SPATS2L expression level was increased with increasing HHT resistant indexes. However, the expression characteristics and prognostic factors of AML remain unknown. We explored the prognostic significance of SPATS2L in AML patients from two independent cohorts. SPATS2L was highly expressed in AML-M5 and intermediate-/high-risk AML patients. AML-M5 has a higher recurrence rate than other types of leukemia and has a poor therapeutic effect [25]. These indicated AML patients with high SPATS2L expression had poor prognosis. The multifactor analysis confirmed that high SPATS2L was an independent poor prognostic factor for OS, EFS, and RFS in AML for the first time Increased SPATS2L expression had stronger prognostic significance and a higher HR for RFS in AML patients, indicating that SPTATS2L might be a better predictor of relapse. Relapse is still a common scenario in AML treatment. 40–50% of younger patients and the great majority of elderly patients will relapse, and the five-year OS is less than 20% after relapse [26]. Minimal residual lesion (MRD) testing by flow cytometry was used to trace AML relapse [27, 28]. However, there is a lack of specific markers for predicting AML relapse. Our results indicated that SPATS2L might serve as an ideal predictor of RFS in the future.

According to data gathered via the NCBI AceView tool, the function of SPATS2L is still unknown. In the present study, the expression level of SPATS2L decreased after CR, indicating SPATS2L may be a candidate therapeutic target for AML. It was confirmed by SPATS2L knockdown using shRNA. Growth inhibition and apoptosis promotion were observed after SPATS2 KD in AML cells. We also found AML cells with SPATS2L KD could induce differentiation. These results indicated SPATS2L could affect AML cell behavior in multiple aspects. By analyzing the RNA sequence, we discovered that high levels of SPATS2L may activate the JAK/STAT pathway. After silencing SPATS2L, the essential proteins in JAK/STAT signaling pathway were also decreased, leading to the inhibition of STAT3 and STAT5 phosphorylation. High expression and constitutive activation of JAK2, STAT3, and STAT5 in AML blasts have been confirmed [22, 29]. JAK-STAT inhibitors, such as STAT3 inhibitors or STAT5 inhibitors, revealed an anti-leukemia effect for *deno* or relapsed/refractory AML [30–32]. The present study also found that the chemokine signaling pathway was suppressed after SPATS2L KD. Cytokine-cytokine receptor interaction was upstream of many pathways in the chemokine signaling pathway, such as JAK/STAT signaling and MAPK signaling pathways. Therefore, SPATS2L may be a promising therapy marker for AML, influencing different pathways, especially JAK/STAT pathway. There are some limitations in the present study. We failed to reveal the regulatory mechanism of SPATS2L in AML more accurately because SPATS2L is currently a molecule whose function is unclear. Its function and protein structure need to be shown in the future.

Three chemotherapy drugs, Ara-c, IDA, and HHT, did not affect the expression of SPATS2L, indicating that these three drugs may have a poor effect on SPATS2L high expression patients. These results also explained why AML patients with high SPATS2L expression had a poor prognosis. Furthermore, inhibition of SPATS2L expression enhanced the apoptosis-promoting effect of these three traditional chemotherapeutic drugs on THP-1 cells. Compared to other AML cell lines, THP-1 shows innate HHT resistance. These findings indicated that targeting SPATS2L may be a promising therapeutic option for treating Ara-c, IDA, and HHT resistant AML patients. Drugs targeting the SPATS2L or JAK/STAT pathways may reverse the chemotherapy resistance. Due to the lack of drugs targeting SPATS2L, we could not test the synergistic effect of SPATS2L targeting inhibitors and AML chemotherapy drugs.

## Conclusion

Here, we suggest SPATS2L is a promising marker for evaluating AML and an effective candidate target for AML treatment. Although the results are promising, the current study had several limitations. First, this study only clarified the prognostic significance and mechanism of SPATS2L in AML pathogenesis. We also found SPATS2L may partly induce drug resistance, and SPATS2L knockdown could reverse HHT resistance. But the underlying mechanism requires further investigation. Second, follow-up studies are needed to identify specific drugs targeting SPATS2L and reveal whether SPATS2L mediates AML chemotherapy resistance (such as Ara-c, IDA) and the in-depth drug resistance mechanism.

## Supplementary Information

**Additional file 1: Figure S1.** Protein localization and expression of SPATS2L in AML. **Figure S2.** The prognostic significance of SPATS2L for AML through quartile survival analysis. **Figure S3.** The changes of cell cycle and differentiation were analyzed by flow cytometry after SPATS2L KD in the AML cell line. **Figure S4.** The Global changes of gene expression between SPATS2L KD and SCR cells, and between SPATS2L high group and low group AML patients were detected by RNA sequencing. **Figure S5.** The top down-regulated pathways after SPATS2L KD were analyzed by KEGG enrichment analysis. **Table S1.** The fold change and prognostic significance of top up genes. **Table S2.** The top down-reguated pathways after SPATS2L KD in AML cells

## Data Availability

All data generated or analyzed during this study are included in this article. Further enquiries can be directed to the corresponding author.

## Abbreviations

AML: Acute myeloid leukemia
DEG: Differentially expressed gene
HHT: Homoharringtonine
SPATS2L: Spermatogenesis associated serine rich 2 like
CR: Complete remission
OS: Overall survival
EFS: Event-free survival
RFS: Relapse-free survival
BM: Bone marrow
PB: Peripheral blood
GWAS: Genome-wide association analysis
RI: Resistant indexes
APL: Acute promyelocytic leukemia
NCG: NOD/ShiLtJGpt-Prkdcem26Cd52Il2rgem26Cd22/Gpt
GSEA: Gene Set Enrichment Analysis
KEGG: Kyoto encyclopedia of genes and genomes
