# Supplementary material for: Spermatogenesis associated serine rich 2 like plays a prognostic factor and therapeutic target in acute myeloid leukemia by regulating the JAK2/STAT3/STAT5 axis
Source: J Transl Med. 2023 Feb 11;21:115. doi: 10.1186/s12967-023-03968-0 (PMC9921581; doi:10.1186/s12967-023-03968-0)
Supplement: Supplementary file 1 — Additional file 1: Figure S1. Protein localization and expression of SPATS2L in AML. Figure S2. The prognostic significance of SPATS2L for AML through quartile survival analysis. Figure S3. The changes of cell cycle and differentiation were analyzed by flow cytometry after SPATS2L KD in the AML cell line. Figure S4. The Global changes of gene expression between SPATS2L KD and SCR cells, and between SPATS2L high group and low group AML patients were detected by RNA sequencing. Figure S5. The top down-regulated pathways after SPATS2L KD were analyzed by KEGG enrichment analysis. Table S1. The fold change and prognostic significance of top up genes. Table S2. The top down-reguated pathways after SPATS2L KD in AML cells [file 12967_2023_3968_MOESM1_ESM.pdf]

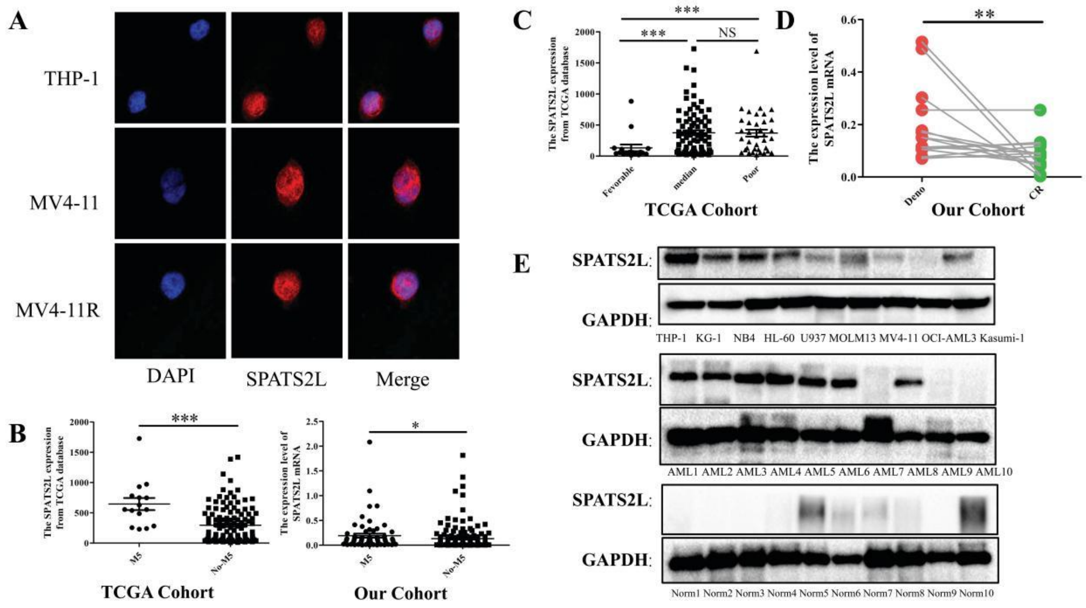

Fig.S1 Protein localization and expression of SPATS2L in AML

A. Protein localization of SPATS2L was analyzed by confocal fluorescence in THP-1, MV4-11, and MV4-11R, repectively. B. Expression level of SPATS2L between AML-M5 patients and No-M5 (not AML-M5 patients) from TCGA data (left) and our data (right). C. The expression levels of SPATS2L among different risk stratifications of AML patients by WHO from TCGA data. D. Expression levels of SPATS2L between newly diagnostic AML patients (*Deno*) and after remission. E. Protein expression level of SPATS2L in AML cell lines (above), primary AML patients (middle), and normal samples (bottom). NS not significance, $P < 0.05$ *, $P < 0.01$ **, $P < 0.001$ ***.

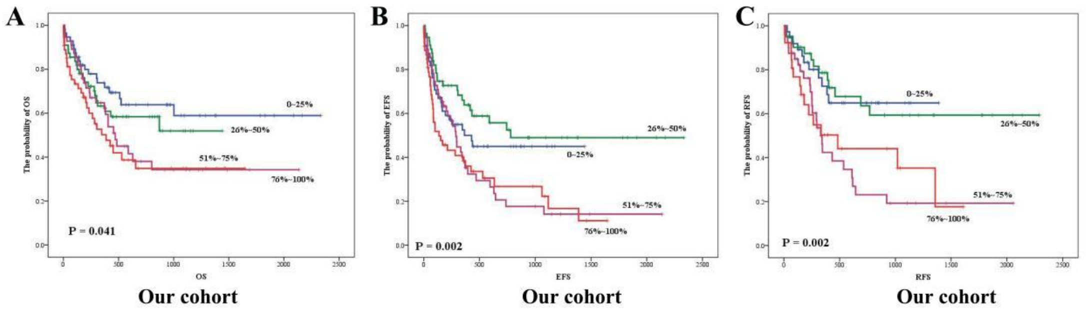

Fig.S2 The prognostic significance of SPATS2L for AML through quartile survival analysis

A. The OS of SPATS2L was calculated by quartile survival analysis in AML patients.

B. The EFS of SPATS2L was calculated by quartile survival analysis in AML patients.

C. The RFS of SPATS2L was calculated by quartile survival analysis in AML patients.

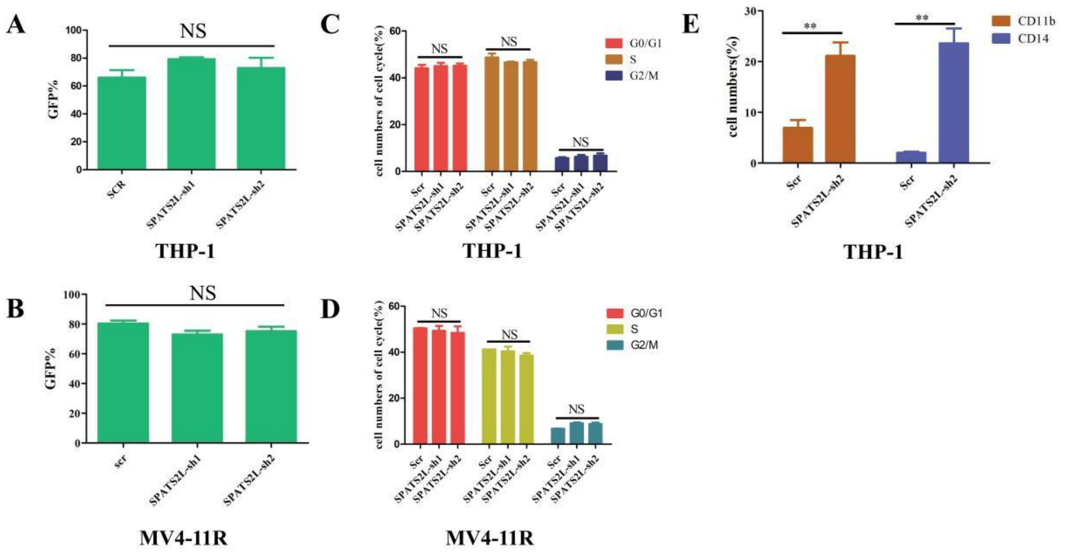

Fig.S3 The changes of cell cycle and differentiation were analyzed by flow cytometry after SPATS2L KD in the AML cell line. A-B After transfected with lentivirus for 72 hours, the transfection efficiency was detected by GFP expression rate using flow cytometr in THP-1 and MV4-11R cells. C-D The cell cycle of THP-1 and MV4-11R transfected with SPATS2L control or KD lentivirus for 72 hours. E. The differentiation antigen CD11b and CD14 were tested by flow cytometry in THP-1 cells after SPATS2L KD.

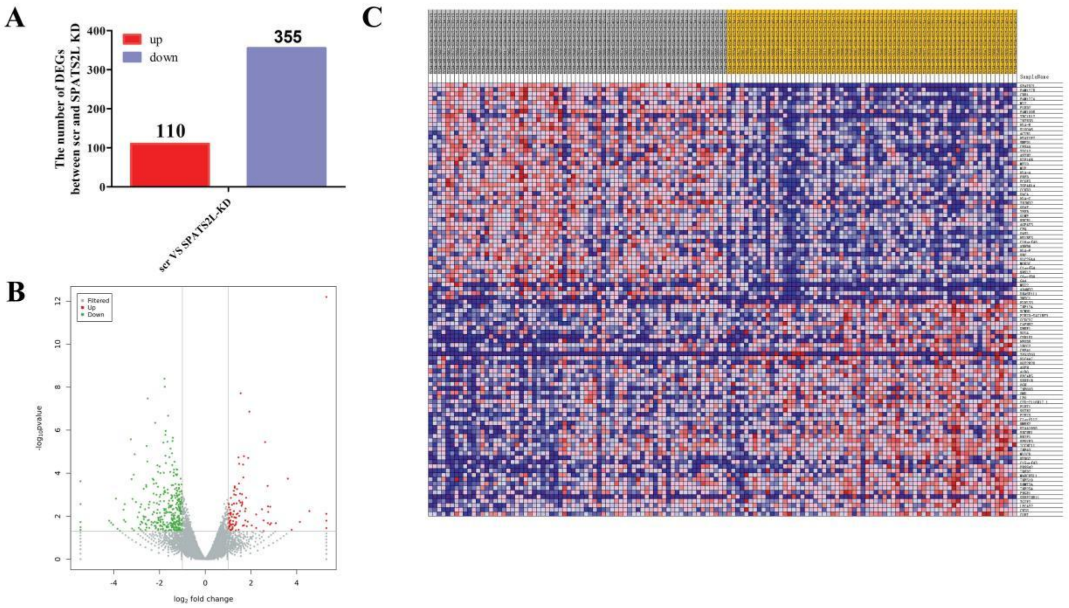

Fig.S4 The Global changes of gene expression between SPATS2L KD and SCR cells, and between SPATS2L high group and low group AML patients were detected by RNA sequencing.

A. The number of up and down DEGs of THP-1 cells with or without SPATS2L KD. B The volcano map of DEGs. C. The heatmap of SPATS2L high group (gray) and SPATS2L low group (yellow) from TCGA database was analyzed by gene set enrichment analysis (GSEA).

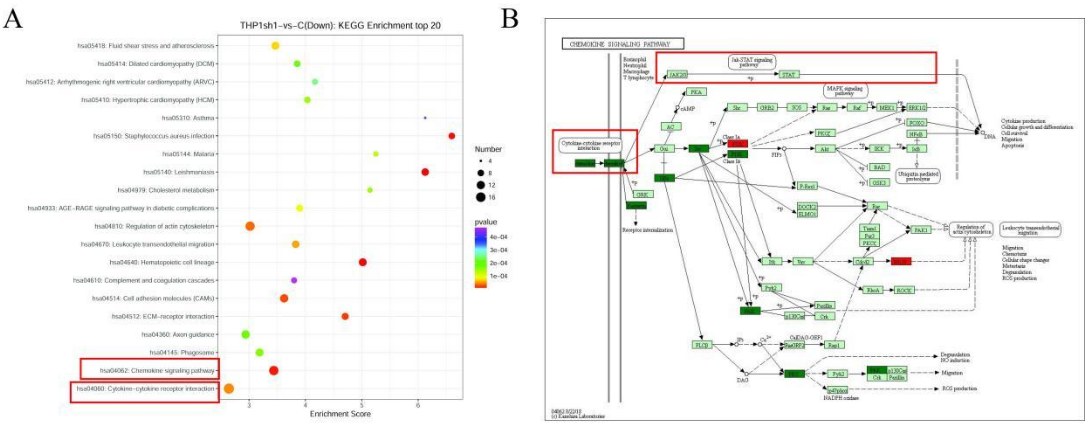

Fig. S5 The top down-regulated pathways after SPATS2L KD were analyzed by KEGG enrichment analysis.

A. The top 20 down pathways were revealed by KEGG enrichment after SPATS2L KD in AML cells. The enrichment score, numbers of genes, P value displayed by bubble chart. Chemokine signaling pathway and Cytokine-cytokine receptor interaction pathway were marked by red box. B. The KEGG map of chemokine signaling pathway. Cytokine-cytokine receptor interaction and JAK/STAT signaling pathway were included in it and were marked by red box.

TableS1: The fold change and prognostic significance of top up genes

Top up gene	Fold change of DEGs			prognostic for AML	P value
	MV4-11R10	MV4-11R30	MV4-11R50		
NAV3	20.6308517	35.41246731	77.46110173	no	0.13
BHLHE22	6.748916117	43.18539129	39.27913471	no	0.79
SERPINB2	35.02477248	20.85048341	32.6449856	no	0.37
KIF21A	12.94415711	19.87342121	26.06486288	no	0.078
CLEC12A	21.34711554	21.28116051	24.26346364	no	0.8
RAB7B	17.64402372	26.66173067	20.17150893	no	0.077
GATA2	20.57241745	24.74179981	19.65319169	no	0.93
CD82	8.728334018	11.19038554	17.8146506	no	0.26
PERP	8.52282635	16.23056206	16.80227635	no	0.76
CXCL8	6.997145411	16.30362699	15.85499872	no	0.77
NPR3	21.16801541	39.7648196	15.5432375	no	0.85
ADGRL3	5.357386651	10.41930583	14.9460154	no	0.36
MGAM2	6.928016496	8.118416247	13.37648428	no	0.34
PRL	9.297237813	31.72440924	12.53243021	no	0.62
SERPINB10	10.05966573	9.013064455	12.043988	no	0.32
MYBPH	6.942322326	17.54906381	11.48346253	no	0.65
ARL4C	20.68077488	12.74709394	11.05023206	no	0.11
SLAIN1	8.938820786	9.657897187	10.6826333	no	0.065
MUC19	6.924430808	15.27400584	10.16116595	no	0.2
TARP	6.323476176	6.959150713	10.01809009	no	0.23
CAV1	10.53422078	10.3534502	9.85376676	no	0.97
MS4A3	5.838933653	6.862003094	9.658080674	no	0.098
SPATS2L	5.34897868	6.498306616	9.570080113	yes	<0.0001
C1orf21	9.031372999	9.4081559	8.787495097	no	0.82
C1orf186	7.290900016	15.27705174	8.451412742	no	0.26
CDK14	5.927419604	8.261518752	8.124764587	yes	0.022
COL24A1	5.272399343	8.051117721	7.844491532	no	0.31
UNC13D	4.87564405	6.744817186	7.818611824	yes	0.029
HAL	4.990725683	7.131995867	6.802762863	no	0.14

TableS2: The top down-reguated pathways after SPATS2L KD in AML cells

ID	term	ListHits	pval	Enrichment_score	Gene
path:hsa05140	Leishmaniasis	10	5.27E-07	6.125106	FCGR2A; NCF2; TNF; HLA-DRA; HLA-DPA1; FOS; PRKCB; ITGAM; ITGB2; CYBB
path:hsa04640	Hematopoietic cell lineage	11	1.59E-06	5.017374	ITGA6; IL7R; CSF1R; TNF; HLA-DRA; HLA-DPA1; CD36; FLT3; ITGAM; CSF2RA; IL3RA
path:hsa05150	Staphylococcus aureus infection	8	2.57E-06	6.596268	FCGR2A; HLA-DRA; HLA-DPA1; C5; SELPLG; ITGAL; ITGAM; ITGB2
path:hsa04062	Chemokine signaling pathway	15	6.54E-06	3.43923	FGR; GNG4; CXCR4; CX3CR1; CCR1; CCR3; CCR2; CCL24; PIK3CG; PTK2; PRKCB; ARRB2; CCL23; SRC; CXCR3
path:hsa04512	ECM-receptor interaction	9	1.83E-05	4.705874	SV2A; ITGA6; FN1; ITGB5; CD36; ITGB7; ITGA7; COL4A1; ITGA11
path:hsa04514	Cell adhesion molecules (CAMs)	12	2.43E-05	3.623302	ITGA6; VCAN; HLA-DRA; HLA-DPA1; SDC2; ITGB7; SELPLG; ITGAL; ITGAM; PECAM1; LRRC4B; ITGB2
path:hsa04810	Regulation of actin cytoskeleton	15	3.36E-05	3.019418	CXCR4; ITGA6; FN1; ITGB5; IQGAP2; PTK2; ITGB7; ITGA7; ITGA11; IQGAP1; ITGAL; ITGAM; ITGAX; SRC; ITGB2
path:hsa04060	Cytokine-cytokine receptor interaction	18	4.52E-05	2.643025	CXCR4; CX3CR1; CCR1; CCR3; CCR2; IL7R; CSF1R; TNF; TNFRSF21; CCL24; ACVRL1; IL16; CCL23; IL27RA; IL2RB; CSF2RA; IL3RA; CXCR3
path:hsa04670	Leukocyte transendothelial migration	10	5.48E-05	3.828191	NCF2; CXCR4; RHOH; PTK2; PRKCB; ITGAL; ITGAM; PECAM1; ITGB2; CYBB
path:hsa05418	Fluid shear stress and atherosclerosis	11	7.24E-05	3.467891	NCF2; GPC1; TNF; VEGFA; SDC2; PTK2; FOS; PECAM1; MAP2K6; THBD; SRC
path:hsa04933	AGE-RAGE signaling pathway in diabetic complications	9	9.49E-05	3.897795	F3; FN1; TNF; VEGFA; VEGFB; COL4A1; PRKCB; THBD; CYBB
path:hsa05144	Malaria	6	0.000125	5.250091	TNF; CD36; SDC2; ITGAL; PECAM1; ITGB2

path:hsa04979	Cholesterol metabolism	6	0.000142	5.145089	SORT1; CD36; LPL; STAR; ABCA1; APOC2
path:hsa05410	Hypertrophic cardiomyopathy (HCM)	8	0.000149	4.035364	SLC8A1; ITGA6; ITGB5; TNF; CACNA2D4; ITGB7; ITGA7; ITGA11
path:hsa04145	Phagosome	11	0.000163	3.18671	FCGR2A; NCF2; TUBA4A; ITGB5; HLA-DRA; HLA-DPA1; CD36; ITGAM; CD209; ITGB2; CYBB
path:hsa04360	Axon guidance	12	0.00021	2.940051	SEMA4A; SEMA4C; CXCR4; EPHB1; EPHB3; UNC5A; EPHA1; PTK2; NRP1; EFNA2; SRC; PARD6B
path:hsa05414	Dilated cardiomyopathy (DCM)	8	0.000212	3.853999	SLC8A1; ITGA6; ITGB5; TNF; CACNA2D4; ITGB7; ITGA7; ITGA11
path:hsa05412	Arrhythmogenic right ventricular cardiomyopathy (ARVC)	7	0.000249	4.168475	SLC8A1; ITGA6; ITGB5; CACNA2D4; ITGB7; ITGA7; ITGA11
path:hsa05310	Asthma	4	0.000414	6.125106	FCER1G; TNF; HLA-DRA; HLA-DPA1
path:hsa04610	Complement and coagulation cascades	7	0.000473	3.799116	F3; C5; SERPINA1; ITGAM; ITGAX; THBD; ITGB2
